# Personality traits and suicide attempts with and without psychiatric disorders: analysis of impulsivity and neuroticism

**DOI:** 10.1186/s12888-017-1453-5

**Published:** 2017-08-15

**Authors:** Bo Bi, Wei Liu, Die Zhou, Xu Fu, Xiaoxia Qin, Jiali Wu

**Affiliations:** The First Affiliated Hospital, China Medical University, 155 North Nanjing Street, Shenyang, 110001 Liaoning, China

**Keywords:** Personality traits, Suicide attempt, Psychiatric disorders,Impulsivity, Neuroticism

## Abstract

**Background:**

There is a critical need for empirical data concerning the association of personality traits and attempted suicide with and without psychiatric disorders in mainland China. The objective of the present study is to provide such data by determining the prevalence of psychiatric disorders and analyzing the levels of impulsivity and neuroticism among people who have attempted suicide, and to examine the association between these personality traits and suicide attempt in people with or without psychiatric disorders.

**Methods:**

We administered self-reported tests and clinical interviews to 196 people who have attempted suicide who were admitted to a hospital emergency room or our psychiatric settings after a suicide attempt.

**Results:**

One hundred and fifty-six subjects (79.6%) met the criteria for Axis I disorders and eleven (6.6%) met the criteria Axis II personality disorders. Those who have attempted suicide who did not have psychiatric disorders exhibited a greater degree of background characteristics (e.g., high lethality, more interpersonal conflicts and more alcohol use), lower levels of suicidality (suicide risk, depressive symptoms) and differences of personality traits (e.g., more impulsive and less neuroticism) as compared to those who do have psychiatric disorders. Profile differences existed even after control for the stressful life event.

**Conclusion:**

Our findings suggest that some personality traits differ between people who have attempted suicide depending on whether or not they have psychiatric disorders. Based on these findings, investigating the impact of personality traits on suicidal behavior in therapeutic settings would provide critical data to improve patient treatment and outcomes.

## Background

Factors contributing to suicidality are diverse and complex. Suicide is a global phenomenon in all regions of the world; in fact, 78% of global suicide occurred in low-and middle-income countries in 2015 [1]. The annual suicide rate was 11.4 per 100,000 people worldwide in 2012, and was associated with approximately 804,000 suicidal behaviors [2]. Additional studies are clearly needed to identify effective treatment and prevention strategies for suicidal behavior disorder, as underlined in the fifth edition of the Diagnostic and Statistical Manual of Mental Disorders (DSM-5) [3].

The term “suicide attempt” describes any nonfatal suicidal behavior, such as intentional self-inflicted poisoning, injury, or self-harm [4]. One or more prior suicide attempts is a major predictor of death by suicide; thus, current suicide prevention techniques focus on monitoring the rates, demographic pattern, and methods of suicide attempts [5]. Psychopathological and neurobiological risk factors are also becoming increasing foci of studies aimed at understanding the complex etiologies of suicide attempts. Among the various predictors of suicidal behaviors, presence of psychiatric disorder seems to be an important determinant for such behaviors [6–8]. The risk of suicidal behavior has been associated with schizophrenia, bipolar disorder, alcohol use disorders, major depressive disorder, obsessive compulsive disorder and personality disorders [9–14]. While the vast majority of attempted and completed suicides occur in people with psychiatric disorders [15], most psychiatric disorder patients do not experience suicidal ideation. Furthermore, a substantial proportion of people exhibiting suicidal behaviors do not exhibit psychiatric disorder diagnostic criteria [16]. Thus, further characterizing factors conferring suicide risk in individuals with or without psychiatric disorders are needed to better define means of prevention and treatment.

The stress-diathesis model is presently an optimal tool for suicidal behaviors. In this model, select personality traits are highly influential in patient inclination toward suicidal behavior. These traits are seen more often in at-risk patients, notwithstanding any diagnosis of a psychiatric disorder. Patients predisposed to suicide often feel defeated or entrapped, which leads to suicidal behavior [17]. Notably, an individual’s predisposition to social perception influences self-identification as failed or trapped. Using this model, a subset of personality traits have been identified as relevant characteristics potentially conferring suicide risk, including impulsivity, aggression, neuroticism, psychoticism, introversion, anxiety, anger, suspiciousness, hopelessness, self-criticism, perfectionism, guilt, resentment and irritability [14, 18]. Impulsivity/aggression has been demonstrated as an important suicide risk factor, and is also typically present in patients with conduct disorder, personality disorders, substance use disorders and bipolar disorders [19]. It is logical to hypothesize that impulsivity and suicidal behavior are correlated, because impulsive individuals might be more likely to enact a suicidal ideation [20]. The personality trait neuroticism, which is demonstrably associated with both depression and unstable mood, is also a focus of suicide research. Bowen et al. demonstrated that neuroticism could predict depression and suicidal thoughts [21]. Interestingly, some personality traits could be predictive of psychiatric disorders. For example, neuroticism has been described as linking anxiety and depressive disorders. However, the underlying mechanisms that explain why some personality traits confer greater risk for suicidality and/or psychiatric disorders are not fully understood. Previous studies reported that personality disorders associated with affective lability and impulsivity had a higher prevalence among those who attempted suicide than other types of personality disorders [22, 23]. Thus, it is necessary to study and identify different aspects of personality traits, such as impulsivity or neuroticism, in people with or without psychiatric disorders who have attempted suicide.

Because psychiatric disorders and select personality traits are significant suicidality risk factors, their relationships remain a complex issue. Thus, studying these relationships in the context of attempted suicide may help to define mechanisms underlying attempted suicide to better determine the suicide risk of an individual patient. Some researchers have pointed out that the patient’s objective state comprises life events and present psychiatric state (depression or psychosis, whereas a propensity toward suicidal behavior is conferred by the patient’s subjective state, including personality traits and suicidal ideations [24]. Most such studies have been limited to patients in Europe, North America or Asia Pacific regions other than mainland China, thus limiting their applicability to Chinese patients [25]. In Chinese patients, only one study has analyzed the association between DSM-IV Axis II personality disorders and attempted suicide [26], finding low prevalence of such disorders both in the local population and among people with suicidal behavior. The authors suggested that studying personality traits may provide deeper insight into the etiology of suicide in China. Thus, comparing differences in personality traits in people with and without psychiatric disorders who have attempted suicide in mainland China is important.

Most studies that have interrogated the roles of personality traits in suicide attempts have not focused on specific personality traits and their associations with psychiatric disorders, opting instead to focus more broadly. The present study aims to delve deeper into specific personality traits while controlling for covariates like environmental stressors. While we aim to validate prior findings, we also seek to explore the associations between personality traits and attempted suicide and compare personality trait (impulsivity and neuroticism) contributions to suicidality in the context of psychiatric disorder presence using a representative sample of patients who were evaluated in emergency services or psychiatric department of a large tertiary academic hospital in China. Thus, the objective herein is to collect expanded data on the roles of impulsivity and neuroticism in suicidality in a Chinese population, for which little data exists. More specifically, we describe herein the prevalence of Axis I and Axis II mental disorders in individuals who have had a recent suicide attempt, and assessed the validity of using these personality traits as independent risk factors. We hypothesized that patient personality traits would differ between patients with or without psychiatric disorders, and that those differences are independent of severe stressful life events.

## Methods

### Selection criteria

This study enrolled patients treated in the emergency department of The First Hospital of China Medical University in Shenyang (population, 7 million; Liaoning Province, northeastern China) for self- or nonself-reported suicide attempts. A trained, bachelor’s-level research assistant interviewed all patients admitted to the either the emergency department or to our psychiatric clinic via liaison psychiatry service within 72 h of a suicide attempt. Data, in turn, was collected via direct interview by the research assistant, from medical records, and from interviews with co-resident family members. Exclusion criteria included the following: 1) aged under 14 years; 2) inability to give informed consent to or understand the study procedures; 3) presence of one or more significant medical impairments (such as dementia) that would limit study participation; or 4) inability to provide a contact person to ensure follow-up assessment participation. The Institutional Review Board of China Medical University approved this study, and written informed consent was obtained from all participants.

### Participants

We enrolled patients from June 2014 to March 2016; during this timeframe, 363 patients were admitted to the target emergency departments after suicide attempts (we defined a “suicide attempt” as any self-inflicted injury with a demonstrated intent of death) were treated at the target emergency rooms. Two hundred and ninety-four patients met the inclusion criteria. Of these patients, 29 were not interviewed (principally due to discharge prior to research team arrival), 67 declined to participate, 2 provided insufficient data for further analysis, and 196 (66.7%) completed full assessments. A total of 196 patients participated in the study, 70 of whom were identified as having attempted suicide multiple times and 126 had attempted suicide only once prior to study enrollment. Mean participant age was 38.9 years (standard deviation (SD), 16.7; range, 14–86), 65% of participants were female, and 95% were of Han Chinese ethnicity.

### Clinician-administered and self-report measures

The study employed a comprehensive interview that included several components and required approximately 2 h to complete. This questionnaire assessed patient sociodemographic data (including age, sex, employment and marital status, residential location, and educational level), detailed description of the relevant suicide attempt (including method, alcohol use during the self-harm behavior, length of time the patient considered suicide before self-harm), prior suicide attempts, and any history of suicidality among friends or family members. In this study, we classified suicide attempt methods as low-lethal and high-lethal. High lethality includes pesticide ingestion, hanging, jumping and carbon monoxide poisoning. By contrast, low-lethal methods included barbiturate or benzodiazepine poisoning and using sharp instruments for self-injury.

The Beck 19-item Scale for Suicidal Ideation was used to evaluate the intensities of patient behaviors, attitudes, and specific plans to commit suicide [27]. This scale includes 19 items, each of which comprise three selections that are scored 0–2 to indicate the intensity of suicidal tendency.

Clinicians administered the 17 item Hamilton Rating Scale for Depression to rate depression severity; this scale is considered reliable with construction and predictive validity [28, 29].

The Chinese translation of the Structured Clinical Interview for DSM-IV Axis I Disorders (SCID), fourth edition, which is demonstrated to be reliable and valid, was used to make diagnoses [30, 31]. This version included “not otherwise specified” (NOS) illness categories for patients with symptoms that were clinically-significant and presented with social dysfunction, but did not present with full criteria to be diagnosed with any specific disorder (relatively common in China). This SCID version also allowed multiple diagnoses to be recorded and ranked by clinical importance. The four psychiatric researchers who performed diagnoses for this study were fully trained to use the SCID via a 4-week course. Interrater reliability after training was excellent (intraclass correlation coefficient = 0.9), which was assessed via 16 taped interviews of different patient types.

Holmes and Rahe assigned a number of “Life Crisis Units” (LCUs) to different life events for subsequent evaluation and treatment [32]. The idea behind this approach is to evaluate patient life events using the LCU rating and LCU table, totaling LCUs for events occurring in the year leading up to the assessed suicide attempt.

Aggression and impulsivity were determined using a self-report questionnaire using the Barratt Impulsiveness Scale (BIS). The 25-item BIS-11-CH assesses aggression and impulsivity in three areas: inability to plan (failure to consider long-term consequences of actions), cognitive impulsivity (rapidly shifting attention and intolerance of complexity), and motor impulsivity (acting rashly). The BIS-11-CH has an internal consistency of 0.83, which was deemed acceptable for this study [33].

Participant personality traits were assessed via the Eysenck Personality Questionnaire, which is a yes-no questionnaire consisting of 12-item indices for psychoticism [P], extraversion [E], and neuroticism [N], as well as a 12-item lie (L) scale [34]. A high P scale score indicates withdrawal, indifference to others, and hostility. A high N scale score indicates emotional instability demonstrated by depression, irritability, anxiety, and frequent complaints of physical discomfort. A high E scale score indicates extraversion, optimism, and sociability. A high L scale score indicates a greater proclivity towards hiding true information. The clinical and behavioral assessment was shown in Table 1.

**Table 1:** Overview clinical and behavioral assessment

Scale	Reference	Measure	Items	Remarks
SCID		15 major adult Axis I diagnostic categories,Axis II disorder and suicidality according ICD 10 and DSM IV		Semi-structured interview
HAM-17	Hamilton 1969	Depressive symptoms	21 items, 5 point scale rating from 0 to 4 overall score: >23: very severe; 19–22: severe, 14–18: moderate, 8–13: mild, 0–7: no depression	Multiple choice questionnaire expert rating
SSI	Beck et al.,1979	Suicidal ideation; 3 dimensions: active suicidal desire, specific plans for suicide, passive suicidal desire	19-items; 3 point scale rating from 0 to 2; range of score: 0–38, higher scores indicate greater suicidal ideation	Semi-structured interview
BIS	Barratt 1965	Impulsivity; 3 dimensions: non-planning, motor impulsiveness, attentional impulsiveness	30-items; 4 point rating from 1 to 4 Overall score reflects the intensity of impulsiveness	Self-rating scale evaluation of lasting personality markers
EPQ	Eysenck HJ, 1985	Personality; 4 dementions: psychoticism, extraversion, neuroticism, lie	88 items require a yes-no response	Assessing the personality traits of participants
LCUT	Holmes and Rahe, 1985	Life events in the past year of an individual’s life	Score of 300+: At risk of illness. Score of 150–299: Risk of illness is moderate (reduced by 30% from the above risk).Score < 150: Only have a slight risk of illness	Assess a patient’s risk of stress-related illnesses

*SCID* Structured Clinical Interview for DSM-IV Axis I Disorders, *HAM-17* Hamilton Rating Scale for Depression

*SSI* Scale for Suicide ideation, *BIS* Barratt Impulsivity Scale, *EPQ* Eysenck Personality Questionaire

*LCUT* Life Crisis Units

### Statistical analyses

SPSS 17.0 for Windows (SPSS Inc., Chicago IL, USA) was used for all statistical analyses, with *P* = 0.05 (two-sided) indicating statistical significance for all tests. The primary goal of this study was to compare personality trait differences in people with and without psychiatric disorders who have attempted suicide in three areas: 1) background characteristics (i.e. demographic, family, and psychosocial histories); 2) the prevalence of psychiatric disorders; 3) psychopathology (personality traits including impulsivity and neuroticism) differences.

Chi-square analyses were used to compare dichotomous variables (in case of categorical variables), and multivariate analyses of variance (ANOVA) were used to compare continuous variables. ANOVA was conducted for clusters of continuous variables: scores on suicide ideation, n suicide risk, the Hamilton depression scale, Eysenck Personality Questionnaire (including psychoticism [P], extraversion [E], neuroticism [N] and lie (L) scale, and BIS (non-planning, motor impulsiveness, and attentional impulsiveness). An analysis was performed in which Life Crisis Units (LCUs) were used as a covariate to preclude the influence of a stressful life event.

Logistic regression was used to determine factors associated with a psychiatric diagnosis among participants; thus, the dependent variable was the presence of any psychiatric diagnosis. Independent variables were scores on suicide ideation, suicide risk, HAM-17, EPQ and BIS. The Wald statistic was used to determine statistical significance, and Gaussian approximation to the log likelihood of the rate was used to determine 95% confidence intervals. Associations between depression symptoms and personality trait variables were examined using Pearson’s correlation.

## Results

### Background characteristics

Among suicide attempt cases, 85.2% (*N* = 167) had a diagnosis of psychiatric disorder (156 Axis I mental disorders and 11 Axis II mental disorders). As shown in Table 2, comparisons between suicide attempts in patients with and without psychiatric disorders did not yield significant differences in gender, marital status, living situation, or employment status. Thus, we conclude that any observed differences in this study cannot be due to these sociodemographic characteristics. The people who have attempted suicide without psychiatric disorders were younger than those with mental disorders (χ^2^ = 7.07; *P* = 0.03).

**Table 2:** Demographic and Clinical Characteristics of Suicide attempters with and without psychiatric disorders

Characterisics	Psychiatric disorders				Analysis	
	Presence of axis I psychiatric diagnosis		No axis I psychiatric diagnosis		Chi-Square Test	
	*N* = 167	85.2%	*N* = 29	14.8%	χ^2^(df = 1)	*p*
Age group					7.07	0.03
14–18	10	6.0	1	3.4		
18–60	126	75.4	28	96.6		
60+	31	18.6	0	0		
Gender					1.71	0.19
Female	113	67.7	16	55.2		
Male	54	32.3	13	44.8		
Marital status					1.21	0.55
Married	85	50.9	15	51.7		
Never married	52	31.1	11	37.9		
Divored/widowed/Sperated	30	18.0	3	10.3		
Living situation					0.18	0.67
Living with family	137	83.0	25	86.2		
Solitary	28	17.0	4	13.8		
Employment Status					1.47	0.69
Employment	57	34.1	7	24.1		
Unemployment	16	9.6	3	10.3		
Student	51	30.5	9	31.0		
Housewife	43	25.7	10	34.5		
High lethality	76	45.5	24	82.8	13.7	0.001
Friends or acquaintances history of suicide attempt	5	3.0	0	0	0.89	0.35
Family history of suicide attempt	4	2.4	1	3.4	0.11	0.74
Interpersonal conflict at the time of episode	63	37.7	24	82.8	20..30	0.001
Alcohol use at the time of episode	21	12.6	11	37.9	11.72	0.003
Less than 2 h of planning	46	27.6	15	53.3	6.89	0.03

Regarding the method of suicide, high-lethality was significantly greater in people who have attempted suicide without psychiatric disorders than in those with psychiatric disorders (χ^2^ = 13.7; *P* = 0.001). Patients who attempted suicide and do not have psychiatric disorders had more interpersonal conflict before the suicide attempt (χ^2^ = 20.3; *P* = 0.001), more alcohol uses at the time of the episode (χ^2^ = 11.7; *P* = 0.003), and less than 2 h between suicidal ideation and action (χ^2^ = 6.9; *P* = 0.03).

### Psychopathology

To compare differences in participants who attempted suicide with and without psychiatric disorders, multivariate analysis of variance was performed on psychopathology data. Suicide risk scores were significantly greater in people with psychiatric disorders who have attempted suicide than in those without psychiatric disorders. Similarly, Hamilton scale depression ratings were significantly higher for people with psychiatric disorders who have attempted suicide than in those without. Furthermore, EPQN scores were also higher in people with psychiatric disorders who have attempted suicide than in those without psychiatric disorders. However, scores on BIS “Non-planning,” BIS “Motor impulsiveness” and BIS overall score were also significantly higher in people without psychiatric disorders who have attempted suicide than for those with psychiatric disorders. Importantly, as shown in Table 3, nearly all differences that we tested between these groups were significant, even after controlling for stressful life events using Life Crisis Units (LCUs) via multivariate ANOVA. These data suggest that elevated psychopathology among patients with psychiatric disorders who attempt suicide does not reflect greater proportions of people with psychiatric disorders in more stressful life events who have attempted suicide.

**Table 3:** Multivariate analysis of variance of EPQ and BIS scales

Dependent variable	Presence of psychiatric diagnosis	No psychiatric diagnosis	F	*p*
Score on suicide idea	39.84	33.10	2.41	0.09
Score on suicide risk	41.40	31.45	7.58	0.001
HAM-17	21.49	9.24	53.80	0.001
EPQP	60.74	57.93	1.31	0.27
EPQE	60.26	61.21	2.24	0.11
EPQN	55.86	47.43	7.15	0.001
EPQL	39.94	36.55	3.26	0.52
BIS “Non-Planning”	27.18	31.45	5.83	0.003
BIS “Motor Impulsiveness”	18.16	21.00	7.74	0.001
BIS “Attentional Impulsiveness”	13.28	12.69	0.91	0.41
BIS Overall Score	58.62	65.14	5.26	0.006

A covariate: *LCUs HAM-17* Hamilton Rating Scale for Depression, *BIS* Barratt Impulsivity Scale, *EPQ* Eysenck Personality Questionaire psychoticism [P], extraversion [E], neuroticism [N] and lie (L) scale

### Prevalence of psychiatric disorders

Table 4 shows the prevalence of Axis I mental disorders and full-criteria Axis II personality disorders as reported by family members or by the participant directly. Axis I disorder criteria were met in 156 participants (79.6%) and Axis II criteria were met in 11 (6.6%). The most common of these in people who attempted suicide was mood disorder, followed by psychotic disorders, anxiety disorders, personality disorders, substance-related disorders and mental retardation.

**Table 4:** Prevalence of psychiatric diagnosis, personality disorders among suicide attempt

	Suicide attempters	Suicide attempters
	N	%
No axis I psychiatric diagnosis	29	14.8
Presence of axis I psychiatric diagnosis	156	79.6
Mood disorders	105	53.6
Major depression	73	43.7
Bipolar	14	8.4
Dysthymia	12	7.2
Depression disorder NOS	6	3.6
Anxiety Disorder	22	13.2
General anxiety disorder	11	6.6
Phobia	1	0.6
Anxiety disorder NOS	2	1.2
Obsessive compulsive disorder	5	3.0
Stress-related disorder	3	1.8
Psychotic disorders	22	13.2
Schizophrenia	20	12.0
Schizophreniform disorder	1	0.6
Psychotic disorder due to general condition	1	0.6
Substance-related disorders	4	2.4
Somatoform disorders	2	1.2
Mental Retardation	1	0.6
Any Personality disorders	11	6.6

### Result of logistic regression analysis and Pearson’s correlation analysis

Next, multivariate logistic regression analysis was used to examine factors associated with a psychiatric diagnosis (the dependent variable) among people who have attempted suicide, which identified the following independent predictors: a higher Hamilton depression rating score (OR, 1.26; 95% CI, 1.26–1.83), a higher Eysenck Personality Questionnaire score (psychoticism; OR, 1.10; 95% CI, 1.00–1.19), and a lower Life Crisis Unit score (OR, 0.99; 95% CI, 0.97–1.00; Table 5). We demonstrate that both the EPQ subscale and BIS overall score exhibit low correlation to HAMD (Table 6).

**Table 5:** Result of logistic regression analysis: 29 suicide attempts without psychiatric disorders and 156 suicide attempts with psychiatric disorders as a dependent variable

95% CI for Exp(B)						
Independents	B	S.E	Wald	Exp(B)	Lower	Upper
EPQP	.090	.044	4.241	1.095	1.004	1.193
HAMD	.415	.096	18.664	1.515	1.255	1.829
LCUT	−.015	.007	4.454	.985	.971	.999
Constant	−15.030	6.955	4.669	.000		

*EPQ* Eysenck Personality Questionaire psychoticism [P], extraversion [E], neuroticism [N] and lie (L) scale

**Table 6:** Correlations of EPQ and BIS to HAMD (*N* = 196)

Scale	Pearson’s correlation coefficient	p for two-sided significance
EPQP	−.044	.541
EPQE	−.323	.000
EPQN	.278	.000
EPQL	.256	.000
BIS “Non-Planning”	−.217	.002
BIS “Motor Impulsiveness”	−.137	.056
BIS “Attentional Impulsiveness”	.012	.871
BIS Overall Score	−.169	.018

*BIS* Barratt Impulsivity Scale, *EPQ* Eysenck Personality Questionaire

## Discussion

This study enrolled a series of individuals who had attempted suicide who were subsequently admitted to general hospitals in Shenyang, China. We independently administered a comprehensive interview – including a structured psychiatric diagnostic examination – to co-resident family informants or the patient directly. We investigated the personality traits of impulsivity/aggression and neuroticism in Chinese patients who attempted suicide. Stressful life events were controlled for as covariates. We also compared differences in clinically meaningful characteristics between patients who attempted suicide and who did or did not present with psychiatric disorders.

### Main findings

In the present study, patients who had attempted suicide differed significantly depending on the presence of mental disorders in five main dimensions: background characteristics, suicidal risk, levels of depression, aggression/impulsiveness, and neuroticism. These differences may be due to the existence of two different people who have attempted suicide and they form a different dimension. Building on our previous studies [16, 35, 36], we hypothesized that key differences are extant in the personality traits of people with and without psychiatric disorders who have attempted suicide in a mainland Chinese population. This hypothesis is strongly supported by our data. As compared to people with psychiatric disorders who have attempted suicide, those without psychiatric disorders exhibited a greater degree of background characteristics that were more likely to be high-lethality, more interpersonal conflicts before the suicide attempt, and more alcohol use at the time of the suicide attempt.

People with psychiatric disorders who have attempted suicide displayed greater suicide risk and higher levels of depression. Also associated with suicidal behavior were higher levels of impulsivity and aggression only in patients without psychiatric disorders; suicidal behavior was associated with higher levels of neuroticism in patients with psychiatric disorders. Profile differences existed even after controlling for stressful life events. Additionally, no significant differences were observed in age, gender, education, living situation, employment status or marital status, suggesting that these sociodemographic traits do not account for the differences observed between study groups.

### Personality trait differences between people with and without psychiatric disorders who have attempted suicide

Personality traits are endemic to patients and affect situational and emotional perception, decision making and, consequently, behavior. Thus, personality folds into the stress-diathesis model for suicidal behavior [37]. Personality traits can therefore influence a patient’s proclivity to react suicidally in individual situations that are known to affect suicidal behavior. The magnitude and robustness of our observed differences between groups in the present study support the hypothesis that neuroticism and impulsiveness differ in subpopulations with suicidal behavior, such as those with and without psychiatric disorders. These distinctions may help clinicians to better address patient needs and prevent suicide.

Impulsivity and neuroticism are personality traits that are commonly associated with suicidal behavior [38, 39]. Neuroticism is a stable personality trait in which patients exhibit a predisposition to experience mental breakdown in response to stress, and those individuals scoring high for neuroticism are more likely to experience anxiety, worry, fear, anger, frustration, envy, jealousy, guilt, depressed mood, and loneliness. Our investigation shows that the people who have attempted suicide who suffer from mental disorders present with significantly higher levels of neuroticism, which is consistent with a prior report [40]. One prior study reported that alcoholic patients who attempted suicide exhibited more introversion and neuroticism as assessed via the EPQ scale [41]. Furthermore, neuroticism is typically higher in adults who have attempted suicide, particularly in those with affective disorders [42, 43]. Another study also showed that patients aged 75 and older who have attempted suicide scored higher for neuroticism and lower for extroversion than controls [44]. Nonetheless, some studies have demonstrated diminishing significance in personality trait differences when depressive symptoms are taken into account [45]. Our results suggest that neuroticism is a stable trait, considering that both neuroticism and psychoticism were only weakly correlated to depression severity.

The presence of impulsivity/aggression has previously been identified as a factor for suicidal behavior [46]. Because our study demonstrated higher scores on the BIS scale in patients without psychiatric disorders who attempted suicide, it is tempting to hypothesize that these patients are more prone to impulsive behaviors like suicide. However, our results differ from those reported for high-income countries where a patient’s impulsivity is a diathesis risk factor for suicide attempts by mediating the impact of an Axis I disorder, especially in those exhibiting a major depressive disorder [47]. However, our findings are consistent with those describing the association between impulsivity and suicidal behavior in China: both trait and state impulsivity have been demonstrated to strongly predict suicide attempt in Chinese patients [48, 49]. However, individuals with these traits, even when extreme, often begin experiencing difficulties during early adulthood, which do not significantly affect overall social or occupational function. Thus, we conclude that, although prominent impulsivity is clearly associated suicidal behavior risk, it does not rise to the level of a psychiatric disorder as defined by the DSM-IV [26].

People with mental disorders who have attempted suicide presented with higher levels of depressive symptoms in the present study, a finding consistent with prior reports demonstrating persistent depression following a suicide attempt [50]. We found that, in patients without psychiatric disorders who exhibited impulsive traits, suicidality was associated with recent interpersonal conflict, alcohol use at the time of episode, and less than 2 h of suicidal ideation, which is consistent with findings from the Chinese psychological autopsy study [48]. However, these findings may not be accounted for by participant population or methodological differences in the Chinese autopsy study, as only patients who had completed suicide were included and the study employed a case-control psychological autopsy design.

### Strengths and limitations

A major strength of the present study is our patient population, which consisted of individuals who had attempted (not completed) suicide and were admitted to an urban hospital emergency room or our psychiatric setting. We were also able to interrogate relationships between two suicide risk domains. Our relational analysis between psychiatric and personality factors in suicide risk will help to more precisely define at-risk groups to better design individualized preventative and therapeutic interventions, and we assert that the methodology employed in this study can be extended to additional mental health outcomes. Data interpretation was greatly facilitated by using a personality inventory with previously-established associations with both Axis I and Axis II disorders. Because we employed cross-sectional methodology with people who have attempted suicide that included self-reported measures, one limitation is that the possibility remains that depression severity influenced patient responses to personality measures. This limitation would best be addressed by prospective studies of personality vulnerability to future suicide attempt.

## Conclusion

Consistent with other studies, we report that neuroticism is a risk factor for suicidal behavior in people with psychiatric disorders. Similarly, impulsivity is a suicide attempt risk factor for people without psychiatric disorders. We conclude that a reliable, valid and feasible method to assess the severity of personality psychopathology should be developed and employed in mainland China to define relationship between personality and suicidal behavior. Suicide risk is mediated by personality factors such as impulsivity and neuroticism, which can provide a basis for developing patient population-specific interventions in patients based on the presence of psychiatric disorders.
